# Molecular responses of radiation-induced liver damage in rats

**DOI:** 10.3892/mmr.2014.3051

**Published:** 2014-12-04

**Authors:** WEI CHENG, LEI XIAO, AIMUDULA AINIWAER, YUNLIAN WANG, GE WU, RUI MAO, YING YANG, YONGXING BAO

**Affiliations:** Department of Oncology, The First Affiliated Hospital of Xinjiang Medical University, Urumqi, Xinjiang 830011, P.R. China

**Keywords:** radiation-induced liver damage, signaling pathway, transforming growth factor-β1, nuclear factor-κB, connective tissue growth factor

## Abstract

The aim of the present study was to investigate the molecular responses involved in radiation-induced liver damage (RILD). Sprague-Dawley rats (6-weeks-old) were irradiated once at a dose of 20 Gy to the right upper quadrant of the abdomen. The rats were then sacrificed 3 days and 1, 2, 4, 8 and 12 weeks after irradiation and rats, which were not exposed to irradiation were used as controls. Weight measurements and blood was obtained from the rats and liver tissues were collected for histological and apoptotic analysis. Immunohistochemistry, reverse transcription quantitative polymerase chain reaction (RT-qPCR) and western blot analysis were performed to measure the expression levels of mRNAs and proteins, respectively. The serum levels of alanine aminotransferase, aspartate aminotransferase and alkaline phosphatase were increased significantly in the RILD rats. Histological investigation revealed the proliferation of collagen and the formation of fibrotic tissue 12 weeks after irradiation. Apoptotic cells were observed predominantly 2 and 4 weeks after irradiation. The immunohistochemistry, RT-qPCR and western blot analysis all revealed the same pattern of changes in the expression levels of the molecules assessed. The expression levels of transforming growth factor-β1 (TGF-β1), nuclear factor (NF)-κB65, mothers against decapentaplegic homolog 3 (Smad3) and Smad7 and connective tissue growth factor were increased during the recovery period following irradiation up to 12 weeks. The expression levels of tumor necrosis factor-α, Smad7 and Smad4 were only increased during the early phase (first 4 weeks) of recovery following irradiation. In the RILD rat model, the molecular responses indicated that the TGF-β1/Smads and NF-κB65 signaling pathways are involved in the mechanism of RILD recovery.

## Introduction

Due to technical advancements, the application of radiation therapy (RT) in treating hepatic tumors is rapidly increasing. However, the frequent association of RT with concurrent liver cirrhosis is a major challenge in radiotherapy. Irradiation of the non-tumor compartment of the liver may cause cell damage, changes in laboratory assessments and/or clinical signs of liver dysfunction. This is termed radiation-induced liver disease, typically emerging between 4 and 8 weeks after the completion of RT and is accompanied by fatigue, rapid weight gain and ascites (1). In the majority of cases, the course of the disease is stable or transient, however, certain patients develop overt liver insufficiency and treatment-associated mortality (2). No pharmacological therapy is currently available to relieve radiation-induced liver disease and it is important to develop techniques to minimize the toxicity or to identify the toxicity early using biomarkers. Radiation-induced liver damage (RILD) has not been fully investigated in parallel with its clinical development, the pathophysiology of RILD remains to be elucidated and systemic investigation of the biomarkers of RILD has not been performed (3). The present study observed alterations in the expression levels of certain molecules, which may be involved in the pathogenesis of RILD, the potential mechanisms underlying RILD and potential targets for its treatment.

## Materials and methods

### Animals

Male Sprague-Dawley (SD) rats (6-weeks-old, weighing 220±10 g) were used. The rats were housed in the animal breeding house at Xinjiang Medical University, (Urumqi, China) and were maintained in a 12 h light-dark cycle at a constant temperature and humidity. The care and use of the laboratory animals were based on the Guidelines and Regulations for the Use and Care of Animals provided by the Ministry of Science and Technology of the People’s Republic of China. The present study complied with the Principles of Laboratory Animal Care (NIH publication No. 85-23, revised 1985), the Office for Protection from Research Risks Public Health Service Policy on the Humane Care and Use of Laboratory Animals (revised 1986) and the U.S. Animal Welfare Act. The procedures were approved by the ethics committee of Xinjiang Medical University (permit no. IACUC-20121122004).

### RILD model

The 36 SD rats were anesthetized by intraperitoneal injection of ketamine and xylazine (60 and 10 mg/kg, respectively; Jiangsu Henrui Medicine Co., Ltd, Lianyungang, China), prior to irradiation with 6 MV photons at a dose of 300 cGy/min in one fraction to the right upper quadrant of the abdomen (2×2 cm) using a Varian Clinac CX accelerator (Varian Medical Systems, Inc., Palo Alto, CA, USA). The one-time total dose of irradiation was 20 Gy. The rats were then sacrificed by decapitation 3 days and 1, 2, 4, 8 and 12 weeks after irradiation. At each time point, six rats were sacrificed and their liver tissues and blood samples were harvested for analysis. Rats, which were not exposed to irradiation were used as controls.

### Evaluation of liver injury by serum analysis

To evaluate liver injury following irradiation, the weight of the rats were monitored at each time point. The serum levels of alanine aminotransferase (ALT), aspartate aminotransferase (AST), alkaline phosphatase (ALP), total bilirubin (TB) and direct bilirubin (DB) were measured to assess liver function. The serum levels of hyaluronic acid (HA), laminin (LN), type III procollagen (PCIII) and type IV collagen (IV-C) were measured to assess liver fibrosis.

### Histological analysis

Liver sections of ~0.5×0.5 cm were collected for histological analysis by hematoxylin and eosin (H&E; Tianjing Zhiyuan Chemical Agents Co., Ltd, Tianjing, China) and Masson’s trichrome (MT; cat. no. MST-8003/8004; Maixin Biotech Co., Ltd., Fuzhou, China) staining. The extent of liver fibrosis and the assessment of the histological features were performed by two pathologists in a blinded-manner. The area of liver fibrosis was quantified using a microscope (Olympus CX41, Olympus, Tokyo, Japan) equipped with a CCD camera by computer-assisted image analysis using Meta-Morph software version 6.06r (Universal Imaging Corporation, Downingtown, PA, USA).

### Apoptotic analysis

The levels of apoptosis were assessed in the liver sections using terminal deoxynucleotidyl transferase dUTP nick end labeling (TUNEL) with a TUNEL test kit (cat. no. MK1020; Boster Co., Ltd., Wuhan, China). Briefly, the liver sections were digested with proteinase K and incubated with 0.2% Triton X-100 in phosphate-buffered saline (PBS)-Tween 20 (ZSGB-Bio Co., Ltd, Beijing, China) for 30min. The sections were then washed twice using PBS-Tween 20 (2 min each), prior to incubation with 3% H_2_O_2_ in PBS for 10 min at 4°C to block endogenous peroxidase activity. The labeling was performed according to the manufacturer’s instructions. Briefly, 18 μl labeling buffer, 1 μl TdT and 1 μl DIG-d-UTP were added to each slide. The slides were put in a box and left at 37°C for 2 h. The slides were subsequently washed with 0.01 M PBS-Tween 20 three times for 2 min. Blocking buffer was added (50 μl/slide) and incubated at room temperature for 30 min. Anti-DIG-Biotin (1:100) was added and slides were incubated in a box at 37°C for 30 min; The slides were subsequently washed three times with 0.01 M PBS-Tween 20 for 2 min. Finally SABC (1:100) was added to the slides (all reagents were purchased from Tianjing Zhiyuan Chemical Agents Co. Ltd). The sections were then incubated with diaminobenzidine (DAB) and counterstained with Gill’s hematoxylin prior to being dehydrated, cleared in xylene (all Tianjing Zhiyuan Chemical Agents Co. Ltd) and placed under coverslips using a xylene-based mounting medium (Poly-Mount xylene; Polysciences, Inc., Warrington, PA, USA). The apoptotic cells were identified by brown staining in the nuclei. Images were captured from 10 randomly selected fields on each slide section at a magnification of ×400 (Leica DM300; Leica Microsystems, Heerbrugg, Switzerland) and, in each field, the apoptotic bodies were expressed as the percentage of 1,000 nuclei.

### Immunohistochemistry

The liver sections (4 μm) were immunohistochemically stained. The primary antibodies used were as follows: Rabbit monoclonal antibodies against transforming growth factor-β1 (TGF-β1; 1:250), nuclear factor (NF)-κB65 (1:100), mothers against decapentaplegic homolog 4 (Smad4) (1:40), Smad3 (1:250), Smad7 (1:30), tumor necrosis factor-α (TNF-α; 1:200) and connective tissue growth factor (CTGF; 1:200), all purchased from Boster Co., Ltd. The secondary antibody used was the Two-Step IHC Detection reagent (cat. no. PV-6001/6002; ZSGB-BIO, Beijing, China). The DAB kit (cat. no. ZLI-9017/9018/9019; ZSGB-BIO) was then used. The immunohistochemical analysis was performed in paraffin sections using a microwave-based (MYE-1870MEG; Haier, Qingdao, China) antigen retrieval technique (4). Following immunostaining, the sections were counterstained with hematoxylin and images were captured using a Leica DM300 microscope and analyzed using Leica Application Suite V3. 35.0 (Leica, Mannheim, Germany). The percentage of the positive staining areas were quantified using Image-Pro plus software (Media Cybernetics, Bethesda, MD, USA).

### RNA extraction and reverse transcription quantitative polymerase chain reaction (RT-qPCR)

The total RNA was isolated from the liver tissues using TRIzol reagent (Roche Diagnostics, Basel, Switzerland) according to the manufacturer’s instructions. RT-qPCR was performed for TGF-β1, NF-κB65, Smad4, Smad3, Smad7, TNF-α and CTGF using a PrimeScript^TM^ one-step RT-PCR kit (Takara Bio, Inc., Shiga, Japan) with GAPDH as an endogenous control and Bio-Rad iQ SYBR Green supermix with Opticon2 (Bio-Rad Laboratories, Inc., Hercules, CA, USA). The primers used were as follows: GAPDH, forward 5′-AGTGCCAGCCTCGTCTCATAG-3′ and reverse 5′-CGTTGAACTTGCCGTGGGTAG-3′; TGF-β1, forward 5′-GTGGCTGAACCAAGGAGACG-3′ and reverse 5′-CAGGTGTTGAGCCCTTTCCAG-3′; TNF-α, forward 5′-ACAAGGCTGCCCCGACTAT-3′ and reverse 5′-CTCCTGGTATGAAGTGGCAAATC-3′; CTGF, forward 5′-TGTGTGATGAGCCCAAGGAC-3′ and reverse 5′-AGTTGGCTCGCATCATAGTTG-3′; NF-κB65, forward 5′-ATCTGCCGAGTGAACCGAAACT-3′ and reverse 5′-CCAGCCTGG TCCCGTGAAA-3′; Smad3, forward 5′-AGGCAGGTCTGGGCTTTATT-3′ and reverse 5′-CGTATCCACAAAGCTGAGCA-3′; Smad4, forward 5′-CACTCGAGGGATCCGAATTC-3′ and reverse 5′-GCGTCGACAAGCTTTCTAGA-3′; Smad7, forward 5′-GAAGTCAAGAGGCTGTGTTGC-3′ and reverse 5′-CAGGCTCCAGAAGAAGTTGG-3′. The oligonucleotide primers for RT-qPCR were purchased from Sangong Biotech Co., Ltd. (Shanghai, China). Relative quantification was performed using the ΔΔCt method [ΔΔCt = ΔCt (treated) - Ct (control)] (5). The ratio of the mRNA expression levels of interest were normalized with the internal control GAPDH.

### Western blot analysis

The protein from the liver tissues were extracted using radioimmunoprecipitation buffer and western blot analysis was performed. The liver tissue samples were homogenized in radioimmunoprecipitation buffer (Thermo Fisher Scientific, Inc., Rockford, IL, USA). The lysates were clarified by centrifugation at 12,000 × g for 10 min and the protein concentrations were determined using a bicinchoninic acid protein assay kit (Thermo Fisher Scientific, Inc.). The samples were mixed with loading buffer and boiled for 5 min at 100°C prior to separation using 10% sodium dodecyl sulfate-polyacrylamide gel electrophoresis followed by transferring onto polyvinylidene fluoride membranes (Roche Diagnostics). The membranes were blocked in Tris-buffered saline (TBS) containing 5% non-fat milk and 0.1% Tween-20 for 1 h at room temperature. The membranes were then incubated at 4°C overnight with the following primary antibodies: Mouse polyclonal anti-TGF-β1 (cat. no. ab92486; 1:20; Abcam, Cambridge, MA, USA), rabbit polyclonal anti-CTGF (1:1,000; Thermo Fisher Scientific, Inc.), rabbit polyclonal anti-NF-κB65 (1:100; Boster Co., Ltd.), rabbit polyclonal anti-TNF-α (1:200; Boster Co., Ltd.), rabbit monoclonal anti-Smad3 (1:100; Boster Co., Ltd.), rabbit monoclonal anti-Smad4 (1:10,000; Boster Co., Ltd.), rabbit monoclonal anti-Smad 7 (1:500; Boster Co., Ltd.) and mouse monoclonal anti-GAPDH (1:5,000; Boster Co., Ltd.). The membranes were then washed three times for 10 min each in TBS containing 0.1% Tween-20, prior to incubation with the corresponding peroxidase-conjugated goat anti-rabbit immunoglobulin G/horseradish peroxidase secondary antibody (PV-6001, ZSGB-Bio Co., Ltd) and washed, as previously. The protein bands were detected using a chemiluminescence detection system (WesternBreeze; Invitrogen Life Technologies, Carlsbad, CA, USA) and autoradiography film (Biomax XAR film; Kodak, Shenzhen, China).

### Statistical analysis

The data are presented as the mean ± standard deviation. The significance of the differences between different groups were determined by one-way analysis of variance, followed by paired-samples t-test using SPSS software version 10.0 (SPSS, Inc., Chicago, IL, USA). A χ^2^ test was performed to compare the ratio data. P<0.05 was considered to indicate a statistically significant difference.

## Results

### Evaluation of liver injury by serum analysis

The weight of the rats was reduced significantly in the 20 Gy irradiation group compared with the untreated group (Table I). This reduction in weight started 2 weeks after irradiation and continued until the end of the study at 12 weeks. The levels of AST, ALT and ALP in the serum were increased significantly in the 20 Gy group from 2 weeks after irradiation to the end of the study. However, no significant changes in the levels of TB and DB (Table II) or the levels of HA, LN, PCIII and IV-C (Table III) were detected in the serum.

### Liver histopathology and TUNEL

**Figure 1:**
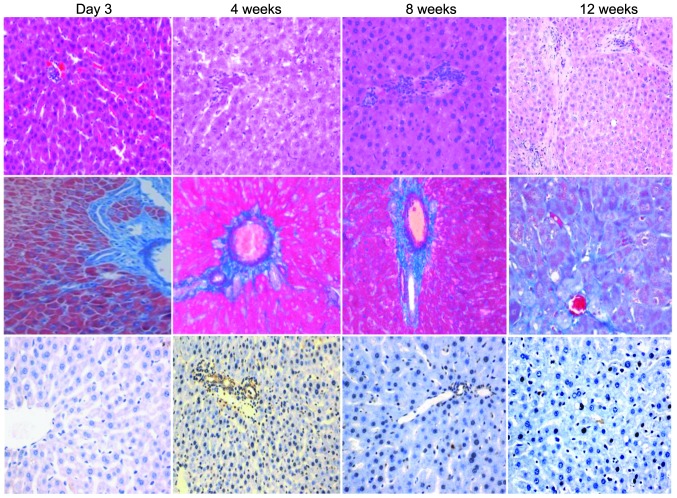
Liver sections stained with H&E, MT or TUNEL, 3 days and 4, 8 and 12 weeks after irradiation. (Top) H&E staining demonstrating the fibrosis proliferation in the liver tissue. Fibrosis of the liver was observed 12 weeks after irradiation. (Middle) MT staining of the distribution of collagen in blue. The staining between the hepatocytes was observed 12 weeks after irradiation. (Bottom) TUNEL staining of the apoptotic cells in brown. Numerous apoptotic cells were observed in the perivenous area 4 weeks after irradiation. (Magnification, ×400). H&E, hematoxylin and eosin; MT, Masson’s trichrome; TUNEL, terminal deoxynucleotidyl transferase dUTP nick end labeling.

The images of the H&E staining demonstrated fibrotic proliferation in the liver tissues. The apparent fibrosis of the liver tissue was observed 12 weeks after irradiation (Fig. 1, top). The MT images revealed that the collagen levels were increased significantly 12 weeks after irradiation, located around the perivenous area and between the hepatocytes (Fig. 1, middle). The apoptotic cells, detected by TUNEL, were observed in the perivenous area 2 and 4 weeks after irradiation (Fig. 1 bottom).

### Immunohistology, RT-qPCR and western blot analysis

**Figure 2:**
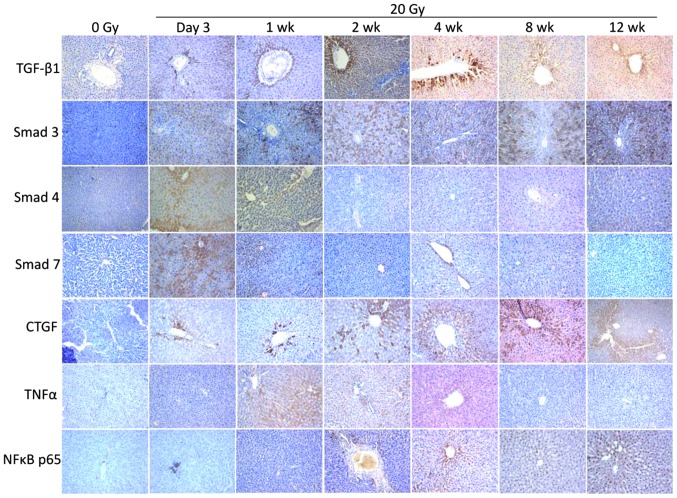
Immunohistochemical staining of liver sections 3 days and 1, 2, 4, 8 and 12 weeks after 20 Gy irradiation. Brown color denotes positivity. The perivenous area was the major area of positive staining. TGF-β1, Smad3, CTGF and NF-κB p65 staining was positive between 3 days and 12 weeks after irradiation. Smad4 and Smad7 staining was positive between 3 days and 1 week after irradiation. TNF-α staining was positive between 1 and 4 weeks after irradiation. (Magnification, ×200). Gy, grays; TGF-β1, transforming growth factor-β1; CTGF, connective tissue growth factor; TNFα, tumor necrosis factor-α, NF, nuclear factor.

**Figure 3:**
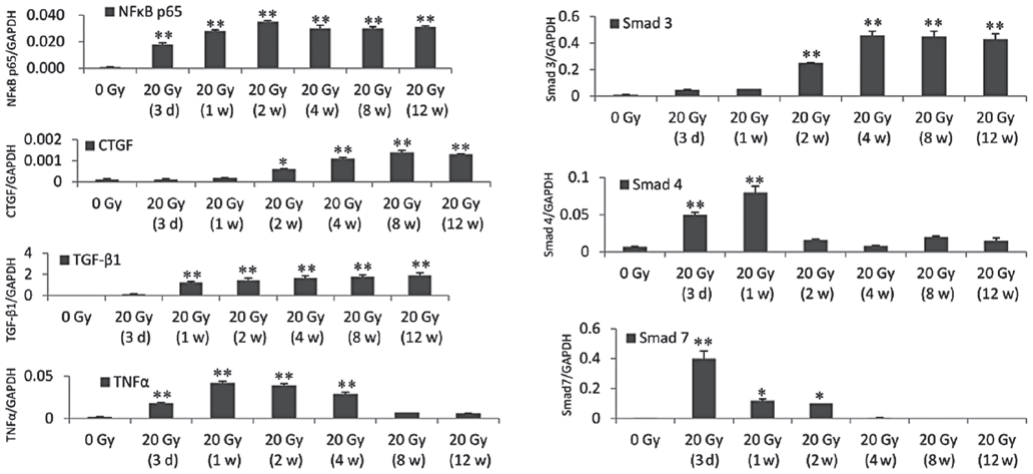
mRNA expression levels were determined using reverse transcription quantitative polymerase chain reaction (ratio of the molecules investigated, vs. GAPDH). The mRNA expression of NF-κB p65 was upregulated between 3 days and 12 weeks after irradiation. The mRNA expression levels were as follows: CTGF and Smad3 were significantly upregulated between 2 and 12 weeks after irradiation. TGF-β1 was significantly upregulated between 1 and 12 weeks after irradiation. TNF-α was significantly upregulated between 3 days and 4 weeks after irradiation. Smad4 was significantly upregulated 3 days and 1 week after irradiation. Smad7 was significantly upregulated 3 days after irradiation and reduced significantly 1–2 weeks after irradiation, however, the expression levels remained higher than that in the control. From 4 weeks post-irradiation, the mRNA expression of Smad7 returned to the control level. ^*^P<0.05, ^**^P<0.001, compared with the control group (0 Gy). CTGF, connective tissue growth factor; TGF-β1, transforming growth factor-β1; TNFα, tumor necrosis factor-α, Smad, mothers against decapentaplegic; NF, nuclear factor.

**Figure 4:**
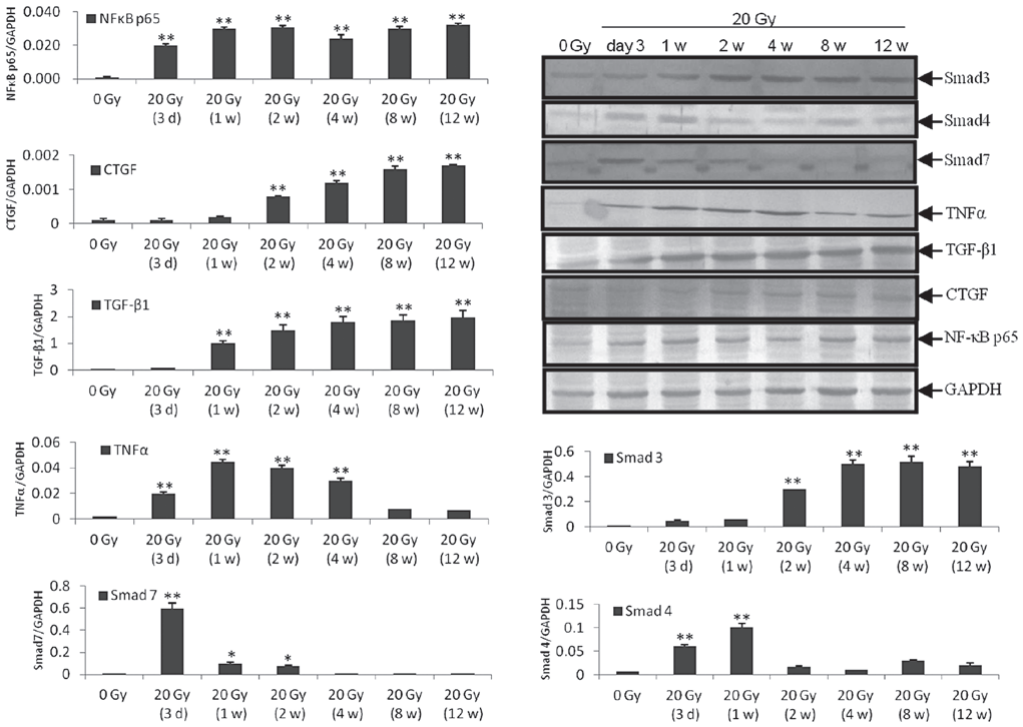
Western blot analysis (ratio of the molecules investigated, vs. GAPDH). Similar to the results obtained using the reverse transcription quantitative polymerase chain reaction, the protein expression levels were as follows: NF-κB p65 was increased between 3 days and 12 weeks after irradiation. CTGF and Smad3 were significantly increased between 2 and 12 weeks after irradiation. TGF-β1 was significantly increased between 1 and 12 weeks after irradiation and TNF-α was significantly increased between 3 days and 4 weeks after irradiation. Smad4 was significantly increased between 3 days and 1 week after irradiation. Smad7 was significantly increased 3 days after irradiation and reduced significantly 1 and 2 weeks after irradiation, however, it remained higher than that in the control. From 4 weeks post-irradiation, the protein expression of Smad7 returned to the control level. ^*^P<0.05, ^**^P<0.001 compared with the control group (0 Gy). Gy, grays; CTGF, connective tissue growth factor; TGF-β1, transforming growth factor-β1; TNFα, tumor necrosis factor-α; Smad, mothers against decapentaplegic; NF, nuclear factor.

The immunohistochemical, RT-qPCR and western blot analysis all revealed the same alterations in the expression levels of TGF-β1, NF-κB65, Smad4, Smad3, Smad7, TNF-α and CTGF (Fig. 2–4). The immunohistochemical analysis demonstrated that the main area of positive staining was the perivenous area. The positive staining of TGF-β1, Smad3, CTGF and NF-κB65 increased between 3 days and 12 weeks after irradiation. Positive staining of Smad4 and Smad7 only occurred 3 days and 1 week after irradiation and positive staining of TNF-α only occurred between 1 and 4 weeks after irradiation (Fig. 2). The RT-qPCR and western blot analysis revealed that the expression level of NF-κB65 was increased 3 days after irradiation and remained at a high level to the 12th week. The expression levels of CTGF and Smad3 were significantly increased at 2 weeks and remained at a high level to the 12th week after irradiation. The expression level of TGF-β1 was significantly increased 1 week after irradiation and remained high to the 12th week after irradiation. The expression level of TNF-α was significantly increased 3 days after irradiation and remained high to the 4th week after irradiation. The expression level of Smad4 was significantly increased 3 days and 1 week after irradiation. The expression level of Smad7 was significantly increased 3 days after irradiation and was reduced significantly 1 and 2 weeks after irradiation, however the expression level remained higher than the control. The expression level of Smad7 returned to the control level 4 weeks after irradiation (Figs. 3 and 4).

## Discussion

RT has become valuable in treating patients with liver cancer, who are unsuitable for surgery or exhibit recurrence following surgery. However, radiation induces hepatic toxicity, which can be fatal (2). RILD is characterized by anicteric ascites and hepatomegaly, isolated elevation in the levels of ALP and/or markedly elevated serum transaminases (1,6,7). In the present study, the levels of AST, ALT and ALP were elevated until 2 weeks after irradiation, which mimicked the clinical situation. The tolerance of normal liver tissues to RT limits the level of RT that can be administered to a patient undergoing cancer treatment (2). Although RILD is a dose-limiting complication, reduced doses may not be sufficient to control tumor growth. Consequently, it may not be possible to cure cancer as a result of the limitations imposed by normal tissue tolerance (8,9).

The tolerance-dose of the whole liver is low and liver damage is observed in 5–10% of patients treated with fractionated doses of 30–35 Gy irradiation (10). Previous studies using RILD rat models have observed different doses causing RILD, between 4 and 60 Gy (11–15). Our preliminary study used 5, 10 and 20 Gy irradiation to establish a RILD rat model. The results revealed that 5 Gy irratiation caused no apparent liver damage and, although RILD wasinduced at 10 Gy, the results were less stable those observed at 20 Gy irradiation, which was used in the present study. This suggested that ≥20 Gy is required to establish a successful RILD model in SD rats. Veno-occlusive disease (VOD) is the most frequently reported histopathological change following human whole liver irradiation (16) and is characterized by perivenous fibrosis, intimal proliferation with concentric endothelial thickening and luminal narrowing by either edematous reticular or collagen fibers (17). Collagen proliferates along the hepatic sinusoids and produces mild congestion in periportal areas. In the present study, H&E and MT staining revealed collagen proliferation and perivenous fibrosis 12 weeks after irradiation, indicating that the VOD in the RILD rat model occurred the at late phase of injury. Collagen proliferation and perivenous fibrosis are responses of the liver to irradiation stimulation (17). The growth factors and cytokines involved in inflammation and immunity may be important in this process. In RILD patients, hepatic stellate cells are activated (18). Stellate cells have multiple functions are involved in the regeneration of hepatocytes and secretion of lipoproteins, growth factors and cytokines, which are important in modulating inflammation and fibrosis (19,20). Of these cytokines, TGF-β has been implicated in subendothelial and hepatic fibrosis in RILD (21,22). TGF-β is produced by numerous inflammatory, mesenchymal and epithelial cells and converts fibroblasts and other cell types into matrix-producing myofibroblasts (23–25). Despite its role in normal wound healing, increased expression of TGF-β1 has been demonstrated in a number of conditions characterized by excessive fibrosis, including chronic hepatitis and glomerulosclerosis (26–30). In addition, effective treatments for these conditions have been observed to reduce the development of fibrosis in the affected organ, with a corresponding decrease in the expression of TGF-β1 (31,32). The present study demonstrated that irradiation of the liver tissue activated TGF-β1 from 1 week after irradiation to the end of the study (12 weeks after irradiation). This indicated that certain inflammatory, stellate, mesenchymal and epithelial cells may be involved in the complex process of radiation-induced liver fibrosis by acting as cellular sources of active TGF-β1. It has been suggested that the irradiation-induced activation of TGF-β1 is rapid (33–35) and prolonged exposure to TGF-β1 stimulates fibrosis and activates the TGFβ1 signal transduction pathway (36,37). The active form of TGF-β1 can then signal through either the Smad-dependent or Smad-independent pathways. The TGF-β1 interactome is highly complex, with several proteins interacting with its transmembrane receptors and signaling proteins (Smads) within the cytoplasm and the nucleus, affecting signaling crosstalk and protein transcription (38,39). There is substantial evidence supporting the importance of TGF-β1 in the development of excessive fibrosis following exposure to radiation in animals and humans (40,41). In addition, certain forms of radiation injury may develop via Smad-independent TGF-β1 signaling (42,43). Following exposure to radiation, reactive oxygen species are produced, which are capable of activating latent TGF-β1 (44,45). The upregulated levels of CTGF observed in the present study suggested that it may be involved in mediating TGF-β1-induced fibroblast collagen synthesis, as described previously (46). In the present study, the mRNA and protein expression levels of TGF-β1 were elevated at the early phase of recovery and were sustained at a high level until the late phase of recovery. Similar changes were observed in the expression levels of Smad3 and CTGF. However, the levels of Smad4 and Smad7 were only elevated in the early phase of recovery. The different time windows observed in the molecular alteration of the TGF-β1/Smads pathway indicate different potential strategies for RILD intervention.

In the present study, the levels of NF-κB, an active transcription factor in the radiation-induced adaptive response (47), were upregulated. A cluster of NF-κB regulated cytokines, including TNF-α, are induced by radiation and contribute to the sensitivity of cells to radiation (48). TNF-α activates NF-κB via receptor activation (49) and regulates the expression of numerous immune and inflammatory response genes (50). TNF-α, which normally induces an acute phase response in hepatocytes, becomes an apoptotic agent (51). In the present study, apoptosis was observed in the liver tissues following irradiation, which was most severe between 2 and 4 weeks after irradiation. However, the mRNA and protein expression levels of TNF-α were increased as early as 3 days after irradiation and were sustained at a high level until the 4th weeks after irradiation. These results are in accordance with a previous report that radiation induces the upregulation of TNF-α and causes hepatocytes to become susceptible to TNF-α mediated apoptosis (52). Previous animal studies have observed that the initial inflammatory reaction following irradiation is not followed by a recovery phase and complete restitution. Instead, progressive liver fibrosis and cirrhosis is regularly observed (53,54). TNF-α is involved in disparate processes, including apoptosis, cell survival, inflammation and immunity and may be an important initial step towards RILD and liver fibrosis (53,54).

In conclusion, numerous cells, mediators and signaling pathways are involved in the initiation and progression of RILD, suggesting multiple complex potential mechanisms are involved in the prevention and treatment of this disease. The present study examined the potential targets of these pathways. The alterations observed in the levels of the molecules and their different time windows provided valuable information for future studies. The results suggested that the mechanisms may involve the early phase inflammation and immunity following irradiation and the late phase of the recovery with fibrosis formation. The NF-κB-mediated radiation response in the early phase requires further investigation and TNF-α may activate NF-κB following irradiation. The TGF-β1/Smads pathway is important for liver fibrosis, with levels of CTGF being upregulated at the late phase of irradiation. In addition, the specific roles of Smad3, Smad4 and Smad require identification. Further studies on these pathways and on the pharmaceutical intervention of these potential targets in the pathway may be valuable for eliminating RILD.

## Figures and Tables

**Table I:** Weight changes in the rats during recovery following irradiation.

Time period	0 Gy group (g)	20 Gy group (g)	P-value
Before irradiation	223.00±4.795	222.00±8.794	>0.05
After irradiation			
3 days	237.18±6.461	216.91±26.27	>0.05
1 week	254.64±30.50	230.73±24.95	>0.05
2 weeks	274.64±23.95	240.73±24.03	<0.05
4 weeks	372.32±12.65	290.12±25.42	<0.05
8 weeks	421.78±11.98	332.75±24.08	<0.05
12 weeks	510.15±23.17	437.80±35.41	<0.05

Gy, Grays.

**Table II:** Analysis of liver function in the rats during recovery following irradiation.

Time period	AST (ng/l)	ALT (ng/l)	ALP (ng/l)	TB (ng/l)	DB (ng/l)
No radiation	88.45±9.83	51.40±4.24	104.45±12.23	0.00±0.00	1.20±0.00
After irradiation					
3 days	74.05±6.32	40.80±8.36	153.46±61.02	0.00±0.00	1.13±0.21
1 week	73.60±0.28	38.25±3.32	241.95±41.65	0.00±0.00	0.85±1.20
2 weeks	117.2±2.55	73.1±61.23	256.7±104.02	0.00±0.00	1.40±0.42
4 weeks	127.98±3.27	75.28±17.43	223.56±17.22	0.00±0.00	1.48±0.29
8 weeks	177.02±15.16	65.34±15.09	228.00±4.24	1.05±0.57	2.33±1.28
12 weeks	245.78±2.36	98.32±15.36	278.12±3.85	2.24±1.56	2.79±2.35
P-value	<0.05	<0.05	<0.05	>0.05	>0.05

Data are expressed as the mean ± standard deviation. P<0.05 indicates a significant difference between the 20 Gy and control groups at each time-point. AST, aspartate aminotransferase; ALT, alanine aminotransferase; ALP, alkaline phosphatase; TB, total bilirubin; DB, direct bilirubin.

**Table III:** Assessment of liver fibrosis in the rats during recovery following irradiation.

Time period	Hyaluronic acid (ng/l)	Type III procollagen (ng/l)	Laminin (ng/l)	Type IV collagen (ng/l)
No irradiation	0-110	0-130	1-130	0-84
After irradiation				
2 weeks	157.65	7.18	53.54	83.21
4 weeks	88.85	8.10	57.15	68.50
8 weeks	111.60	8.57	62.32	75.16
12 weeks	74.61	4.95	60.24	43.89
P-value	>0.05	>0.05	>0.05	>0.05

P>0.05 indicates no significant difference between the 20 Gy and control groups at each time-point.
